# Novel Nanotechnology of TiO_2_ Improves Physical-Chemical and Biological Properties of Glass Ionomer Cement

**DOI:** 10.1155/2017/7123919

**Published:** 2017-05-22

**Authors:** Daniela Dellosso Cibim, Miki Taketomi Saito, Priscila Alves Giovani, Ana Flávia Sanches Borges, Vanessa Gallego Arias Pecorari, Orisson Ponce Gomes, Paulo Noronha Lisboa-Filho, Francisco Humberto Nociti-Junior, Regina Maria Puppin-Rontani, Kamila Rosamilia Kantovitz

**Affiliations:** ^1^Department of Pediatric Dentistry, Piracicaba Dental School, University of Campinas, Piracicaba, SP, Brazil; ^2^Department of Prosthodontics and Periodontics, Division of Periodontics, Piracicaba Dental School, University of Campinas, Piracicaba, SP, Brazil; ^3^Department of Dentistry, Endodontic and Dental Materials, Bauru Dental School, University of São Paulo, Bauru, SP, Brazil; ^4^São Paulo University, São Paulo, SP, Brazil; ^5^Department of Physics, School of Science, State University of São Paulo, Bauru, SP, Brazil; ^6^São Leopoldo Mandic Institute, Dental Research Center, Campinas, São Paulo, SP, Brazil

## Abstract

The aim of this study was to assess the performance of glass ionomer cement (GIC) added with TiO_2_ nanotubes. TiO_2_ nanotubes [3%, 5%, and 7% (w/w)] were incorporated into GIC's (Ketac Molar EasyMix™) powder component, whereas unblended powder was used as control. Physical-chemical-biological analysis included energy dispersive spectroscopy (EDS), surface roughness (SR), Knoop hardness (SH), fluoride-releasing analysis, cytotoxicity, cell morphology, and extracellular matrix (ECM) composition. Parametric or nonparametric ANOVA were used for statistical comparisons (*α* ≤ 0.05). Data analysis revealed that EDS only detected Ti at the 5% and 7% groups and that GIC's physical-chemical properties were significantly improved by the addition of 5% TiO_2_ as compared to 3% and GIC alone. Furthermore, regardless of TiO_2_ concentration, no significant effect was found on SR, whereas GIC-containing 7% TiO_2_ presented decreased SH values. Fluoride release lasted longer for the 5% and 7% TiO_2_ groups, and cell morphology/spreading and ECM composition were found to be positively affected by TiO_2_ at 5%. In conclusion, in the current study, nanotechnology incorporated in GIC affected ECM composition and was important for the superior microhardness and fluoride release, suggesting its potential for higher stress-bearing site restorations.

## 1. Introduction

Glass ionomer cements (GICs) are composed primarily of a calcium fluoroaluminosilicate glass powder and an aqueous solution of a homo- or copolymer acrylic acid [1]. The use of GICs is widespread in dental clinical applications, as luting materials, liners and bases, orthodontic bracket adhesives, core buildups, pit and fissures sealants, and restorative materials [1]. GIC has unique properties including its coefficient of thermal expansion close to the tooth structure, biocompatibility, antimicrobial potential, adhesive strength, and anticariogenic capability [2–5]. A recent meta-analysis study confirmed high survival rates for single surface ART restorations using high-viscosity GIC in permanent and primary teeth over 5 and 2 years, respectively [6]. Conversely, GICs have been reported to present clinical limitations such as low wear resistance, low fracture toughness, low mechanical properties, prolonged setting rate, and high early moisture sensitivity [3, 7]. These limitations might contribute to restoration failure with bacterial proliferation and consequent recurrent caries and/or teeth or restoration fractures, in special multiple-surface ART restorations, which are site restorations with high stress bearing [6, 8]. Efforts have been made to improve GICs' physical and mechanical properties without affecting their biological properties, by the addition of a variety of filler materials including silver amalgam alloy, silver powder, montmorillonite clay [9], zirconia [10], glass fibers [11], hydroxyapatite (HA) [12], bioactive glass particles as prereacted glass ionomer particles, and casein phosphopeptide-amorphous calcium phosphate [13].

Nanodentistry is an emerging area in dentistry and uses nanostructured materials for diagnosing, treating, and preventing oral and dental diseases, relieving pain, and preserving and improving dental health [14]. In addition, nanostructured materials have been shown to present improved properties as compared to its bulk form [15–20]. In particular, TiO_2_ nanostructures have been the subject of intense research due to their chemical stability, nontoxicity, and improvement of mechanical properties in composites and hybrid materials [21]. The majority of the nanotechnology-based studies have focused on assessing its effect on GICs' mechanical performance, and, therefore, the impact of TiO_2_ nanoparticles on GICs biocompatibility remains undetermined, as do the effects of TiO_2_ nanofillers on GICs' physical-chemical properties and their fluoride release capabilities. Not many studies have assessed the impact of TiO_2_ nanofillers on GICs' surface roughness and hardness, or on its potential to interfere with dental biofilm formation and maturation [17, 22]. The structural differences among the various nanomaterials (e.g., nanoparticles, nanotubes, nanowires, nanorods, and nanofilms) also need further investigation. Tubular materials are hollow structures that feature a high surface-to-volume ratio [23]. This property may contribute to improving the reaction/interaction between a device and the surrounding medium, thereby making the system more effective or even suggesting novel reaction pathways [23]. In addition, the importance of the nanomaterials' physical-chemical properties, such as size, shape, and surface characteristics, on the biological effects of the underlying structure should be investigated, since nanotubes present an increased reactivity with dental matrix materials. Therefore, the aim of this investigation was to determine the physical-chemical and biological properties of a conventional GIC (Ketac Molar EasyMix) incorporated with different concentrations of TiO_2_ nanotubes. In the current investigation, the first null hypothesis was that TiO_2_ nanotubes added to a GIC would not significantly impact its physical-chemical properties, and the second null hypothesis was that the incorporation of TiO_2_ nanotubes to a GIC would not affect its biological performance.

## 2. Materials and Methods

### 2.1. Experimental Design

The conventional GIC substitute was Ketac Molar Easymix (3M/ESPE, Maplewood, Minnesota, USA, batches #1433900541, #1523000219, and #1426900658). GIC samples were randomly assigned to four experimental groups based on TiO_2_ concentration levels:* Ketac Molar (KM)* = Control; KM + 3% TiO_2_; KM + 5% TiO_2_; and KM + 7% TiO_2_. The parameters under review were energy dispersion (EDS), surface roughness (SR) and hardness (SH), fluoride release (F), cytotoxicity (MTT) and morphology (SEM), and extracellular matrix (ECM) composition. The study was conducted after approval from the Ethics Committee (protocol #527951/16).

### 2.2. Materials

A conventional GIC, KM [shade A3; powder: Al-Ca-La fluorosilicate glass, 5% copolymer acid (acrylic and maleic acid) (15 g); liquid: polyalkenoic acid, tartaric acid, and water (10 g)], and TiO_2_ nanotubes (particle size ~20 nm and diameters of about 10 nm, synthesized by the alkaline route [24]) in the three different concentrations were used in the study. Briefly, TiO_2_ nanotubes were prepared by mixing 12 g anatase TiO_2_ (Aldrich, 99%) in 200 mL of 10 M NaOH. This mixture was kept at 120°C for 24 h in a Teflon open container, which was placed in a glycerin bath, using a mantle heater for heating. The syntheses were carried out at ambient pressure, where only precursor reagents were subjected to alkaline treatment. After the alkaline treatment, the mixture was washed with 0.1 M hydrochloric acid and deionized water repeatedly to remove the sodium ions. Next, the pH of the solution is adjusted to 7. Finally, the materials obtained were dried in a conventional oven at 200°C for 24 h in air atmosphere [24].

*Samples Preparation*. TiO_2_ nanotubes were weighed using a balance, accurate to 0.0001 g (BEL Engineering; Monza, Milan, Italy), and was added to the GIC's component powder prior to hand mix manipulation. The recommended powder/liquid (P/L) ratio of 1/1 for KM was used in all prepared samples. The preparation was in accordance with the manufacturers' specifications, ISO #7489, at room temperature (23 ± 1.0°C and 50 ± 5% relative humidity). The materials were placed into polyvinyl siloxane disk-molds measuring 2 mm in height × 4 mm in diameter (Express™ XT Light body, 3M/ESPE, Seefeld, Germany, #17412-1) in one increment and pressed for six minutes between polyester matrix (Proben, Catanduva, SP, Brazil, #PR5021) and glass plates. They were covered with a thin layer of petroleum jelly and stored for 24 h at 37°C and 100% humidity.

### 2.3. Physical and Chemical Evaluations

#### 2.3.1. Sample Calculation

In order to determine the sample size (*n*) to be used in the current study, pilot experiments were performed and the level of significance (*Z*_a_) and statistical power (*Z*_b_) was calculated at 95% and 85%, respectively. The difference (*D*) to be detected among groups was stipulated at 0.15, 5, and 0.07 for SR, SH, and F, respectively. The value referring to the standard deviation (sd = 0.12, 0.01, and 0.04, resp., to each test) was obtained after a pilot study. To this value, 20% was added to account for eventual losses. Thus, the minimum to be investigated became 8, 8, and 12 of samples to SR, SH, and F, respectively.

#### 2.3.2. Energy Dispersive Spectroscopy Analyze (EDS)

The EDS analysis was performed using a SEM (JEOL-JSM 5600LV, Tokyo, Japan) equipped with an X-radiation detector EDS (Voyager, Noran Instruments), operated in low vacuum and backscattered electron mode. This EDS equipment contains an ultrathin Norvar window and works with a Windows NT-based (Vantage) digital microanalysis system. The surfaces of disc-shaped samples (2 × 4 mm; *n* = 4) were carbon coated via evaporation of high purity carbon rods (Denton Vacuum Desk II, Moorestown, NJ, USA). The whole area of the sample surface was observed by SEM (JEOL-JSM 5600LV, Tokyo, Japan) set at a magnification of 100x, with a working distance of 20 mm and operated at accelerating voltage of 15 kV. The measurements were performed on four different areas of each sample's surface. They were then measured for their titanium (wt% Ti) content, with results expressed as a percentage by Easy Macro software (Noran Instruments, mod. Vantage v.1.2, Middleton, WI, USA).

#### 2.3.3. Surface Roughness Test (SR)

The surface roughness of samples in each experimental group (2 × 4 mm; *n* = 8) was analyzed with a surface roughness-measuring instrument (Surfcorder SE1700; Kosaka Corp, Tokyo, Japan) equipped with a diamond needle of 2 *μ*m radius. In order to record roughness measurements, the needle was moved at a constant speed of 1.25 mm/s under a 0.7 mN load. The cut-off value was set at 0.25 mm to maximize filtration of surface waviness. Three traces were recorded for each sample at three different locations, parallel, perpendicular, and oblique, to scan all sample area. The surface roughness was characterized by the average roughness (*R*_*a*_), which is the arithmetical average value of all absolute distances of the roughness profile from the centerline within the measuring length.

#### 2.3.4. Surface Hardness Test (SH)

Sample surfaces were polished with 800-grit paper SiC (Extec CORP., Enfield, CT, USA, #1060-524) for one minute (Arotec SA Ind. and Com., São Paulo, Brazil) before testing [25]. SH measurements were made on disc-shaped samples (2 × 4 mm; *n* = 8) using a microhardness tester (HMV-2000, Shimadzu, Kyoto, Japan) with a Knoop diamond under a 50 g load for 10 s. The measurement of the indentation was performed immediately. Three indentations spaced 1 mm from each other were made in the central area of each sample. The arithmetical average was used for subsequent statistical analysis.

#### 2.3.5. Fluoride Release Test (F)

The disc-shaped samples (2 × 4 mm; *n* = 8) were protected with an acid-resistant nail varnish (Colorama, São Paulo, SP, Brazil, #38655) with the exception of the surface underneath the polyester matrix (exposed area). A piece of paraffin dental floss was inserted into the materials during setting time to suspend the samples in separate and different media while waiting for further testing (storage media). Samples were randomly distributed by a lottery and individually immersed in 2 mL of each storage media: (a)* demineralizing solution*, DE (2.0 mM calcium, 2.0 mM phosphate, and acetate buffer 75 mM, pH 4.3); (b)* remineralizing solution*, RE (artificial saliva –1.5 mM calcium, 0.9 mM phosphate, KCl 150 mM, and Tris [tris(hydroxymethyl) aminomethane] buffer 20 mM, pH 7.0). Tubes were kept constantly agitated at 120 rpm, 1.7 Hz (Cientec Model CT 165, Piracicaba, SP, Brazil) at controlled temperature of 25° ± 1°C. Storage media were changed every 24 h. Duplicate aliquots of the solutions were mixed with TISAB III at a ratio of 1 : 0.1 and were analyzed using an ion-selective electrode (Orion 96-09; Orion Research Inc., Boston, MA, USA) and a digital ion-analyzer (Orion EA- 940; Orion Research Inc.). The latter was calibrated previously with various standard solutions (0.065, 0.125, 0.250, 0.500, 1.000, and 2.000 mg of F/mL). The values obtained from each sample at 1, 2, 3, 5, 7, 9, 12, and 15 days were analyzed and divided by the sample area (exposed area = 12.56 mm^2^). The amount of released fluoride (ppm) at different time periods was calculated.

### 2.4. Biological Analysis

#### 2.4.1. Preparation of Samples

KM + 3% TiO_2_ and KM + 5% TiO_2_ groups were chosen for further biological testing as they presented significant improved physical-chemical properties as compared to the control. The materials were hand-mixed in a flow chamber and poured into sterile polyvinyl siloxane disc molds (Express XT Light body, 3M/ESPE, Seefeld, Germany, #17412-1) to provide standard samples 2 mm in height and 8 mm in diameter, in preparation for the cytotoxicity assay (*n* = 3), for cell morphology analysis (*n* = 2), and for ECM analysis (*n* = 3). All the samples were subjected to disinfection via ultraviolet light for 10 minutes. Samples were then maintained in a humidified atmosphere for 24 h until their use.

#### 2.4.2. Cell Culture

Upon informed consent from the patient, human gingival fibroblasts (hGFs) were obtained from a healthy 19-year-old male subject. Gingival tissue was rinsed three times with biopsy media formulated by Dulbecco's Modified Eagle Medium (DMEM) high glucose (Gibco, Thermo Fisher Scientific, Waltham, MA, USA, #11965-065) supplemented with 10% Fetal Bovine Serum (FBS) (Gibco, Thermo Fisher Scientific, #10500-064), penicillin (100 U/mL), and streptomycin (100 mg/mL) (Gibco, Thermo Fisher Scientific, #15240-062). It was enzymatically digested in 2 mL of a solution of DMEM containing 3 mg/mL of collagenase type I (Gibco, Thermo Fisher Scientific, #17018-029) and 4 mg/mL of dispase (Gibco, Thermo Fisher Scientific, #17018-029) for 1 h, at 37°C. The solution containing the digested tissue was then transferred to a sterile 15 mL Falcon tube and centrifuged at 2,500 rpm for 5 minutes. The supernatant was discarded and the pellet was resuspended in 5 mL media and plated in culture flasks, which was then incubated at 37°C in a 5% CO_2_ atmosphere. Once hGFs adhered to the flask, the media were changed to DMEM high glucose with 10% FBS (and penicillin, streptomycin). The hGFs were subcultured until a sufficient number to perform the experiments was reached. The cells from the fourth and fifth passages were used in the experiments.

#### 2.4.3. Analysis of Cell Cytotoxicity by 3-(4,5-Dimethylthiazol-2-yl)-2,5-diphenyltetrazolium Bromide (MTT) Assay

After curing the materials, the disc-shaped samples of experimental groups (*n* = 3) were placed individually into sterile 48-well plates (Corning Costar, Cambridge, MA, USA, #CLS3548). Next, hGFs were seeded (1.5 × 10^4^ cells/well) over the discs in DMEM + 10% FBS and incubated in a humidified incubator at 37°C and 5% CO_2_ for 24 h to allow cell adhesion. For the experimental control, hGFs were seeded at the same density, but with no discs. Growth medium was changed every other day. Cell viability was determined at the time points 6, 24, 48, and 72 h by the MTT assay in accordance with the manufacturer's instructions. Growth medium was aspirated and replaced by 900 *μ*L of DMEM added with 100 *μ*L of MTT (5 mg/mL) (Life Technologies, Rockville, MD, USA, #M6494) and incubated at 37°C and 5% CO_2_ for 4 h, protected from the light. Next, 500 *μ*L dimethyl sulfoxide (DMSO) (Sigma-Aldrich, St. Louis, Missouri, USA, #276855) was added to each well to dissolve the formazan crystals that were produced by the cleavage of MTT salt in the mitochondria of viable cells. Three aliquots of 100 *μ*L from each well were transferred to 96-well plate and the absorbance was determined at 570 nm wavelengths, using an Elisa microplate reader (VersaMax, Molecular Devices, Sunnyvale, CA, USA). The mean average of the three reads of each 96-well plate (Corning Costar, #CLS3550) served as the single value for the 48-well plate. Experiments were performed in triplicate and repeated a minimum of two times, according to ISO 10993-5 (2009) recommendations [26].

#### 2.4.4. Analysis of Cell Morphology by Scanning Electron Microscopy (SEM)

The morphology of hGFs on GIC discs, with or without TiO_2_, was assessed by SEM (*n* = 2) at 6, 24, 48, and 72 h. After curing, the discs were placed on 48-well culture plates (Corning Costar, #CLS3548) before seeding hGFs (10^4^ cells/well) in DMEM plus 10% FBS and antibiotics, which were cultured in an incubator at 37°C and 5% CO_2_ for 24 h. After 24 h, culture medium was replaced for DMEM plus 5% FBS and the cells fixed with Karnovsky's solution (pH 7.4) overnight at 4°C at 6, 24, 48, and 72 h and postfixed in 1% osmium tetroxide in distilled water for 1 h at room temperature. Cells were then dehydrated using a 10-minute series of upgrade ethanol treatments at room temperature, beginning with 35%, 50%, 70%, and 90% and ending with two treatments of 100% ethanol. In addition, the samples were dried in a critical point dryer (Denton Vacuum, mod. DCP-1, Moorestown, NJ, USA) and coated with gold, using a sputter coater (BAL-TEC, mod. SCD 050, Fürstentum, Liechtenstein). The samples were examined in a SEM (JEOL, Mod. JSM5600LV, Tokyo, Japan). The working distance was 30 mm, magnification was 50x, and voltage was at accelerated levels up to 15 kV.

#### 2.4.5. Matrix Formation Analysis by Sirius Red

The Sirius Red/Fast Green Collagen Staining kit (Chondrex, Redmond, WA, USA, #9046) was used to assess the amount of collagenous and noncollagenous protein formation by hGFs that were cultured on control and 3% and 5% TiO2 discs (*n* = 3). After curing, the discs were placed on 48-well plate (Corning Costar, #CLS3548) and hGFs were seeded (5 × 10^4^ cells/well) in DMEM plus 5% FBS, penicillin (100 U/mL), and streptomycin (100 mg/mL) (DMEM + 5% FBS). Next, cells were incubated in a humidified incubator at 37°C and 5% CO_2_ for 24 h to allow cell adhesion. Growth medium was changed every other day with DMEM and 5% FBS and collagenous and noncollagenous ECM contents assessed at days 7 and 14 after plating, according to manufacturer's instructions. Briefly, samples were washed with DPBS (Gibco, #14190- 136) and then fixed with Kahle's fixative solution for 10 minutes at room temperature. Next, the fixative solution was removed and samples were washed in DPBS, immersed in dye solution (200 *μ*L/well) for 30 minutes at room temperature, and washed five times with distillated water. One mL of extraction buffer was added to each well. Aliquots of 100 *μ*L were transferred to a 96-well plate (Corning Costar, #CLS3550) and the absorbance was measured at 540 nm and 605 nm, using an Elisa microplate reader (VersaMax, Molecular Devices, Sunnyvale, CA, USA). Mean values of three reads of each well were considered as a single value per well. Collagen quantity was measured according to the formula specified by the manufacture: collagen (*μ*g) = [OD 540 nm value − (OD 605 nm value × 0.291)]/0.0378; noncollagenous protein (*μ*g) = OD 605 nm value/0.00204.

### 2.5. Statistical Analysis

Original data from all evaluations were analyzed using Shapiro-Wilk normality test, before applying parametric tests. Nonparametric tests were used when variance was not homogeneous. Data for EDS, SR, SH, WS, and SL were analyzed using a one-way ANOVA test, followed by Tukey's post hoc test. Kruskal-Wallis and Friedman tests were also used to statistically compare fluoride release and ECM data, whereas the two-way ANOVA was used to compare MTT data. Statistical analyses were carried out using the SPSS 21 program (IBM Brazil, São Paulo, SP, Brazil) and BioEstat 5.3 (Mamiraua Institute, PA, Brazil), with a statistical significance level of 5%.

## 3. Results

### 3.1. Effect on GICs' Physical-Chemical Properties due to Incorporation of TiO_2_ Nanotubes

**Figure 1:**
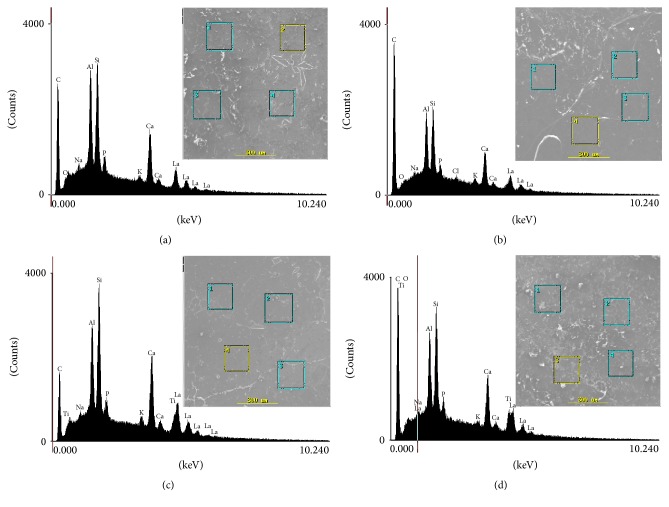
Scanning electron microscopic microphotographs selected areas for the experimental groups (original magnification 100x) and their respective chemical composition by energy dispersive spectroscopy. (a) Control (KM); (b) KM + 3% TiO_2_; (c) KM + 5% TiO_2_; (d) KM + 7% TiO_2_.

Representative SEM micrographs are shown in Figure 1. Data analysis showed no relevant topographical differences among the experimental groups [control (KM), KM + 3% TiO_2_, KM + 5% TiO_2_, and KM + 7% TiO_2_ nanotubes]. The EDS data showed similar dominant proportions of calcium and phosphorus in all materials, whereas titanium was detected only in the 5% and 7% TiO_2_ groups, confirming its incorporation to the GIC. As expected, the highest concentration of TiO_2_ was found in the KM + 7% TiO_2_ group. EDS analysis confirmed the presence, in all experimental groups, of elements typically found in conventional GICs, including aluminum, fluorine, zinc, phosphorus, silicon, strontium, magnesium, and calcium (Figures 1(a)–1(d)).

Table 1 shows the mean values and standard deviation of surface roughness and hardness for all the experimental groups. While there was no significant difference among the groups regarding SR (*p* > 0.05), the SH analysis showed a significantly greater hardness value of KM + 5% TiO_2_ when compared to control (KM) and KM + 7% TiO_2_ groups.

The patterns of fluoride ion release (ppm) are shown in Table 2. For the DE solution, the highest values were registered during the first 48 h followed by a gradual decrease up to the end of the experiment period. For artificial saliva (RE solution), the KM + 7% TiO_2_ and the KM + 5% TiO_2_ groups had similar patterns of fluoride release. Both showed increased values when compared to KM + 3% TiO_2_ (*p* < 0.05). All groups containing TiO_2_ increased fluoride release as compared to control group (KM), except for KM + 3% TiO_2_ (*p* < 0.05). Regardless of which solution was involved or TiO_2_ presence, all experimental groups experienced higher fluoride release rates at their initial periods, with decreased levels overtime.

### 3.2. Effect of Incorporation of TiO_2_ Nanotubes to GIC in Cell Cytotoxicity

**Figure 2:**
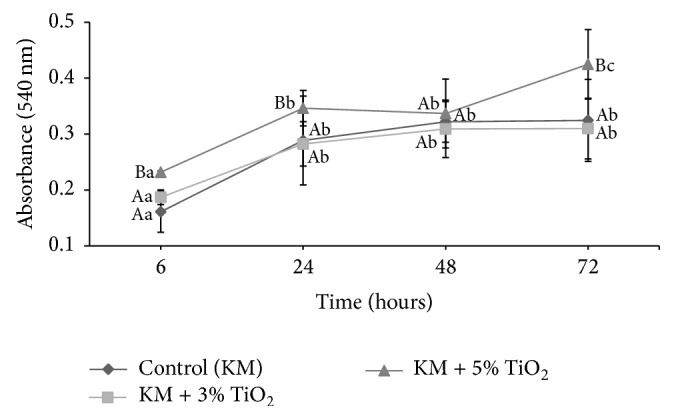
Cell viability of the experimental groups at 6, 24, 48, and 72 h.* Different uppercase and lowercase letters represent intergroup and intragroup differences, respectively, by two-way ANOVA*.

Because of operational issues related to the size of the experiments and availability of reagents, the biological assays focused on the 3% and 5% TiO2 groups as we generally have found that they present more consistent performance on the physical-chemical analysis when compared to the control group. Cell viability, as determined by the MTT assay, is presented in Figure 2. We also found interactions between the two factors investigated: TiO_2_ addition and time (*p* = 0.000). We found statistically significant differences when considering the experimental groups (*p* = 0.000) and time (*p* = 0.000) separately. The highest cell viability rate was observed for the KM + 5% TiO_2_ group, compared to control (KM) and KM + 3% TiO_2_ groups. Overall, GIC resulted in lower viability than cell cultures without GIC, regardless of the incorporation of TiO_2_ nanotubes, and all the experimental groups experienced increased cellular activity overtime.

**Figure 3:**
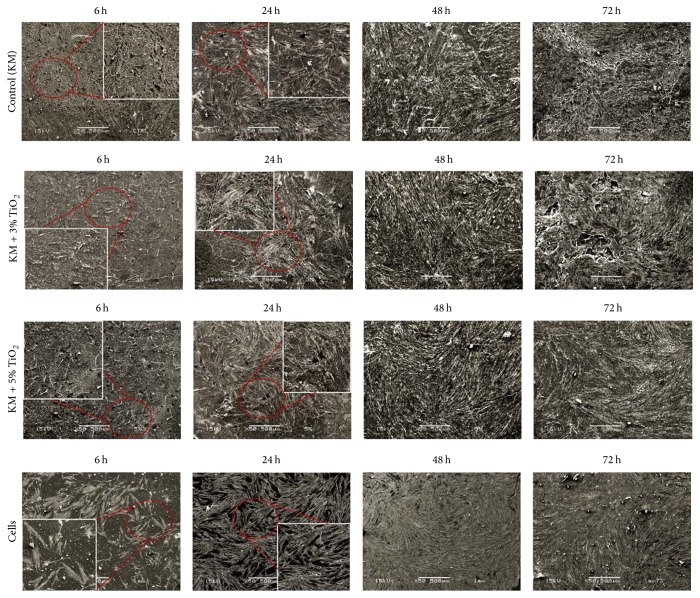
Scanning electron microscopic images of human gingival fibroblast cells adhered to different substrates at 6, 24, 48, and 72 h: control (KM); KM + 3% TiO_2_; KM + 5% TiO_2_ and culture dish (cells). Normal cell morphology was observed for all groups: numerous cells, near confluence, remained adhering to the glass substrate and exhibited an elongated morphology with several thin cytoplasmic prolongations originating from their membrane and formation of layer of cells at 48 and 72 h (SEM original magnification 50x).

SEM analysis revealed a similar pattern for all the experimental groups, featuring elongated flattened cells presenting normal fibroblastic shape. Furthermore, a large number of adhered cells could be seen for all the experimental conditions (Figure 3), which demonstrated that TiO_2_ nanotubes did not affect cell adhesion and growth when compared to GIC alone or no GIC.

Sirius red/fast green assay was used to functionally assess the impact of TiO_2_ nanotubes on the quality of ECM produced by hGFs on GIC substrates (Table 3). Intragroup analysis indicated that ECM collagenous contents were significantly increased, overtime, for KM + 5% TiO_2_ and no GIC substrate (cells) groups. On the other hand, noncollagenous contents were increased overtime on substrate groups formed with KM + 3% TiO_2_ and for no GIC. Furthermore, intergroup analysis showed that GIC substrates, regardless of TiO_2_ addition, resulted in a collagen-richer ECM compared with absence of GIC substrate at days 7 and 14 (*p* < 0.05), whereas GIC substrates decreased the noncollagenous contents at day 14 (*p* ≤ 0.05).

## 4. Discussion

In the present study, it was found that crescent concentrations of TiO_2_ into GIC (3%, 5%, or 7%) did not affect SR, which was always similar to the control group regardless of the TiO_2_ concentration used. These findings can potentially be explained by the fact that nanosized TiO_2_ tubes did not affect the distribution between particles and matrix and the interfacial bonding between particles. These findings are in line with previous studies suggesting that the particle size is a critical factor affecting the material's surface roughness [27, 28] and that the use of nanosized particles may produce favorable surface roughness for dental materials. Therefore, it can be proposed that the addition of TiO_2_ nanotubes to GIC will not negatively affect its performance as far as its surface roughness. In contrast to the current findings, previous studies showed that nanofilled RMGI Ketac N100 exhibited the smoothest surface on the material when compared to conventional GICs [29, 30], consequently suggesting that the superficial roughness is also dependent upon other parameters such as the composition of the underlying material [31].

In the present study, the addition of 5% TiO_2_ to a conventional GIC significantly increased its Knoop microhardness (KHN), whereas 3% and 7% TiO_2_ produced similar results to the control (KM) group. In line with previous studies suggesting that GICs' microhardness may be affected by the ratio of glass particles to polyacid [32], the findings of the current investigation suggest that, indeed, it may be a critical aspect to guide one's decision on the nanofiller concentration for the best microhardness outcomes. Here, it can be speculated that the increased microhardness found for the 5% group resulted from GIC matrix interactions, with TiO_2_ nanotubes at 5% resulting in the ideal proportion of glass particles and acid at the GIC's surface to react with the nanoparticles. As previously suggested [32], in the case of higher levels of TiO_2_, as in the 7% groups, insufficient interfacial bonding of polyacrylic ionomer and the nanotubes to acids will affect GIC's hardness. In contrast, in the present study, at the lower level range, TiO_2_ did not significantly affect the interactions at the GIC's matrix. Although the impact of nanoparticles on GIC's microhardness is controversial and has been reported to be not only dependent on the concentration of TiO_2_ nanotube [17, 22, 29, 30, 32, 33], in the current investigation, data analysis indicates that TiO_2_ nanotubes may affect GIC's superficial microhardness in a concentration-dependent way.

The fact that TiO_2_ nanotubes have the potential to improve GICs surface roughness and hardness is promising, as it may reduce biomechanical degradation of the material. However, additional studies are needed to identify the appropriate conditions for its incorporation. Here, three different TiO_2_ nanotube concentrations were tested and the EDS analysis was used to examine their incorporation to the GIC matrix. The findings of the present study suggest that the incorporation of these nanotubes to the GIC matrix may be dependent upon its initial concentration, as Ti was only detected at the 5% and 7% TiO_2_ groups. Intriguingly, at the concentration of 3%, Ti was not detected by the EDS analysis, and, therefore, it can be speculated that it is due to the fact that such concentration is very close to the resolution of the technique.

Fluoride release is routinely used as a measure of GICs' cariostatic ability. In the current study, the effect of TiO_2_ nanotubes on the ability of a conventional GIC to release fluoride was determined in vitro. It was found that the addition of TiO_2_ nanotubes to a conventional GIC significantly increased its fluoride-releasing pattern. A burst effect of fluoride release was observed during the first day after TiO_2_ nanotubes addition, followed by a gradual decrease until a plateau was reached. A similar pattern has been reported for GIC without the influence of any nanotechnology [12, 13, 25]. It is generally accepted that fluoride release by GIC is controlled by two processes, a relatively fast release from the surface, followed by a longer-term and more gradual release from the bulk of the material due to diffusion through its cement pores and cracks [34]. Whether or not the increased fluoride release promoted by TiO_2_ nanotubes will increase the cariostatic potential of GIC requires further investigations. Similar to previous studies [25, 35], the present investigation found greater amounts of fluoride release in demineralizing (DE) versus remineralizing (RE) solutions. This phenomenon is attributed to a pronounced erosion of the polysalt matrix of the glass ionomer and by increased dissolution of the material in low pH environments [35]. Additional studies should be considered to assist elucidating such aspect.

In dentistry, operative and restorative procedures must attend to biological principles, and, therefore, it is critical to determine how a certain dental material will perform in a biological system. It is generally accepted that in vitro tests, mostly based on cell culture systems, must precede in vivo approaches when testing the biological behavior of dental materials. Thus, the goal of the current investigation was to determine the biological impact of a GIC added or not with TiO_2_ nanotubes using conventional cell culture assays, including MTT, SEM, and the ability of gingival fibroblasts to produce collagenous and noncollagenous extracellular matrix (ECM). Data analysis showed that KM-containing TiO_2_ did not affect cell cytotoxicity by the MTT assay, cell morphology, and spreading by SEM and the capacity of gingival fibroblasts to produce collagenous and noncollagenous rich ECM by the picrosirius red/fast green assay. Instead, when compared to GIC alone, TiO_2_ added GICs presented an increased cellular viability capacity revealed by a higher activity of the dehydrogenase enzyme (MTT) and resulted in similar cellular adherence capabilities featuring physiological morphology with the ability to form comparable collagenous and noncollagenous rich ECM. Although several studies [36–42] have characterized the biological performance of a number of dental materials, including ceramic materials [36], dental composites [37], resin cements [38], and GIC added or not with fillers [37, 39, 40], very limited information is available regarding the impact of TiO_2_ nanotubes on the biological properties of GICs. Garcia-Contreras et al. [41] investigated the biological properties of powdered GICs prepared with TiO_2_ nanoparticles using cancer cells and normal human gingival and periodontal ligament fibroblasts and pulp cell cultures. In line with the findings of the current study, Garcia-Contreras et al. [42] found that GICs impregnated with 3% and 5% TiO_2_ nanoparticles presented an acceptable biocompatibility pattern when compared to nonimpregnated GICs. However, despite the promising results showing that the incorporation of TiO_2_ nanotubes will not negatively impact the biological properties of GICs, caution must be used and further studies considered in order to establish whether such in vitro biological performance of GICs added with TiO_2_ is reproducible and, later, to determine the response pattern of TiO_2_ incorporated dental materials in vivo.

In summary, the available literature gives support to the clinical potential of this concept; however, the future of GIC modified by the incorporation of TiO_2_ nanotubes will strongly depend on the development of additional studies to verify the required conditions to lead us to an outstanding material as far as its physical-chemical and biological performances, which includes, for example, determining the ideal approach to obtain the most homogeneous mixture, as hand mixing issues may be considered one of the limitations of the present study.

## 5. Conclusion

Based on the results of the present investigation, the combination conventional GIC and 5% TiO_2_ nanotubes significantly increased the noncollagenous composition of ECM and was critical for the improved microhardness and fluoride release capability, without affecting surface roughness suggesting its potential for higher stress-bearing site restorations.

## Figures and Tables

**Table 1:** Surface roughness (*R*_*a*_-*µ*m) and hardness (KHN) mean values and standard deviation (SD) for the experimental groups.

Experimental groups	Surface roughness (*R*_*a*_-*µ*m) Mean value ± SD	Surface hardness (KHN) Mean value ± SD
Control (KM)	0.41 ± 0.14 A	81.48 ± 9.87 B
KM + 3% TiO_2_	0.55 ± 0.17 A	105.87 ± 12.71 AB
KM + 5% TiO_2_	0.49 ± 0.07 A	118.25 ± 4.21 A
KM + 7% TiO_2_	0.58 ± 0.16 A	75.13 ± 6.61 B

Different letters represent intergroup significant differences by one-way ANOVA followed by Tukey's test (*p* ≤ 0.05).

**Table 2:** Mean values for fluoride release (ppm F^−^) over time in demineralizing (DE) and remineralizing (RE) solutions for the experimental groups.

	Experimental groups	Time (days)							
		1	2	3	5	7	9	12	15
DE solution	Control (KM)	0.198 ± 0.05 Ba	0.156 ± 0.04 Bab	0.145 ± 0.05 Aabc	0.141 ± 0.05 Abc	0.141 ± 0.05 Bbc	0.124 ± 0.04 Ac	0.159 ± 0.04 Aabc	0.165 ± 0.07 Aab
	KM + 3% TiO_2_	0.298 ± 0.07 Aa	0.233 ± 0.06 ABab	0.199 ± 0.03 Aabc	0.179 ± 0.02 Abc	0.179 ± 0.03 ABbc	0.158 ± 0.03 Ac	0.168 ± 0.04 Ac	0.172 ± 0.04 Abc
	KM + 5% TiO_2_	0.292 ± 0.08 Aa	0.256 ± 0.08 Aab	0.197 ± 0.06 Aabc	0.187 ± 0.05 Ab	0.212 ± 0.06 Aabc	0.155 ± 0.05 Ac	0.213 ± 0.04 Ac	0.213 ± 0.04 Ac
	KM + 7% TiO_2_	0.311 ± 0.08 Aa	0.249 ± 0.09 ABab	0.191 ± 0.05 Aab	0.181 ± 0.05 Abc	0.168 ± 0.05 ABbc	0.154 ± 0.04 Ac	0.171 ± 0.04 Abc	0.164 ± 0.06 Abc

RE solution	Control (KM)	0.049 ± 0.01 Aa	0.03 ± 0.01 Bb	0.033 ± 0.01 Aab	0.027 ± 0.01 Bb	0.030 ± 0.01 Bb	0.032 ± 0.01 Bab	0.031 ± 0.01 Bb	0.031 ± 0.01 Bab
	KM + 3% TiO_2_	0.041 ± 0.01 Ba	0.029 ± 0.01 Bb	0.037 ± 0.01 Aab	0.036 ± 0.01 ABab	0.033 ± 0.01 ABab	0.037 ± 0.01 ABab	0.034 ± 0.01 ABab	0.039 ± 0.01 ABab
	KM + 5% TiO_2_	0.037 ± 0.01 Bab	0.035 ± 0.02 ABb	0.036 ± 0.03 Ab	0.04 ± 0.01 Aab	0.038 ± 0.01 ABb	0.042 ± 0.01 Aab	0.037 ± 0.01 Ab	0.047 ± 0.01 Aa
	KM + 7% TiO_2_	0.067 ± 0.02 Aa	0.047 ± 0.02 Aa	0.043 ± 0.00 Bb	0.041 ± 0.01 Aab	0.039 ± 0.01 Ab	0.044 ± 0.01 Aa	0.045 ± 0.02 Aa	0.044 ± 0.02 Aa

Different uppercase letters in columns indicate intergroup differences by the Kruskal-Wallis test (*p* ≤ 0.05). Different lowercase letters indicate intragroup difference by the Friedman test (*p* ≤ 0.05).

**Table 3:** Mean values (*µ*g/well) and standard deviation (SD) for extracellular matrix (ECM) collagenous and noncollagenous content in cell cultures for days 7 and 14.

	Experimental groups	Day 7 Mean value ± SD	Day 14 Mean value ± SD
Collagenous	Control (KM)	3.35 ± 0.92 Aa	2.81 ± 0.32 Aa
	KM + 3% TiO_2_	2.52 ± 0.31 Aa	2.93 ± 0.60 Aa
	KM + 5% TiO_2_	2.35 ± 0.19 Aa	2.94 ± 0.55 Ab
	Cells	1.41 ± 0.15 Ba	1.98 ± 0.11 Bb

Noncollagenous	Control (KM)	54.9 ± 2.31 Aa	53.3 ± 3.46 Ba
	KM + 3% TiO_2_	65.8 ± 5.53 Ba	54.9 ± 3.89 Bb
	KM + 5% TiO_2_	61.3 ± 4.11 Ba	54.6 ± 4.14 Ba
	Cells	36.9 ± 1.0 Aa	59.7 ± 2.61 Ab

Different uppercase letters indicate intergroup differences by the Kruskal-Wallis test (*p* ≤ 0.05), whereas different lowercase letters indicate intragroup difference by the Friedman test (*p* ≤ 0.05).
